# A prospective cohort study of 14-3-3η in ACPA and/or RF-positive patients with arthralgia

**DOI:** 10.1186/s13075-016-0975-4

**Published:** 2016-04-01

**Authors:** Marian H. van Beers-Tas, Anthony Marotta, Maarten Boers, Walter P. Maksymowych, Dirkjan van Schaardenburg

**Affiliations:** Amsterdam Rheumatology and Immunology Center | Reade, Dr. Jan van Breemenstraat 2, PO Box 58271, 1056 AB, 1040 HG Amsterdam, The Netherlands; Augurex Life Sciences Corp, 887 Great Northern Way, Suite 125-1, North Vancouver, BC Canada; Amsterdam Rheumatology and Immunology Center, VU University Medical Center, PO Box 7057, 1007 MB Amsterdam, The Netherlands; Department of Epidemiology and Biostatistics, VU Medical Center, PO Box 7057, 1007 MB Amsterdam, The Netherlands; University of Alberta, Rheumatic Disease Unit, 116 St and 85 Ave, Edmonton, AB T6G 2R3 Canada; Amsterdam Rheumatology and Immunology Center, Academic Medical Center, PO Box 22660, 1100 DD Amsterdam, The Netherlands

**Keywords:** 14-3-3ŋ protein, Rheumatoid arthritis, Prediction, Anti-citrullinated protein antibodies, Arthralgia

## Abstract

**Background:**

14-3-3η (eta) is a novel serum/plasma protein biomarker involved in the upregulation of inflammatory and joint damage factors. We analysed the association of 14-3-3η with the development of clinically apparent arthritis in a cohort of subjects with arthralgia and positivity for at least one serologic marker: rheumatoid factor (RF) or anti-citrullinated protein antibody (ACPA).

**Methods:**

Measurement of 14-3-3η in plasma collected on entry into the cohort. For this study, 144 subjects with a minimum of 2.5 (median and maximum 5) years of follow-up were available. The relationship between presence and levels of 14-3-3η and development of arthritis was investigated.

**Results:**

Arthritis occurred in 43 (30 %) of the 144 subjects after a median of 15 months. 14-3-3η was detectable up to 5 years before onset of clinical arthritis and was present significantly more often (36 % versus 14 %; relative risk 2.5, 95 % confidence interval 1.2–5.6; *p* = 0.02) and at significantly higher levels (median 0.95 versus 0.28 ng/ml; *p* = 0.02) in subjects developing arthritis compared with those who did not. 14-3-3η levels/positivity and ACPA, but not RF, were univariately associated with the development of arthritis while generalized linear model analysis with RF and ACPA as obligatory factors could not return an incremental benefit with 14-3-3η.

**Conclusions:**

14-3-3η was detectable prior to the onset of arthritis and was associated with arthritis development in arthralgia subjects pre-selected for positivity of RF or ACPA. Its power to predict onset of arthritis independent of ACPA and RF requires a new study in which patients are not pre-selected based on ACPA and/or RF seropositivity.

## Background

The focus on the management of rheumatoid arthritis (RA) is increasingly towards early detection and treatment. Better prediction of the development of RA will potentially allow preventive interventions in these at-risk individuals. Recently, we published a prediction rule for the development of arthritis in rheumatoid factor (RF) and/or anti-citrullinated protein antibodies (ACPA) positive (seropositive) arthralgia patients [1]. Patients could be divided into high, intermediate or low risk categories quite accurately with an area under the receiver operator characteristic (ROC) curve (AUC) of 0.82 at 5 years. However, this is still inadequate for individual patient care – assuming the availability of a preventive intervention; therefore additional biomarkers to improve the predictive arthritis risk algorithm is required.

Several such potential biomarkers were recently described. Examples are anti-carbamylated protein antibodies (anti-Carp) [2], peptidyl arginine deiminase type 4 (anti-PAD-4) [3], and a high interferon gene score [4]. Two of these biomarkers were discovered within the same patient group as we will describe here [2, 4]. Such biomarkers may help to improve prediction, but also offer new insights into the course of events leading to clinical arthritis.

Serum 14-3-3η (eta) is a novel protein biomarker showing potential in predicting radiographic deterioration in early and advanced RA [5, 6]. 14-3-3 proteins belong to a family of seven isoforms known to bind to and regulate the biologic activity of various intracellular proteins [7]. Overexpression of 14-3-3 proteins is associated with worse outcomes in various diseases, such as cancers, neurodegenerative diseases and Creutzfeldt-Jakob's disease. The 14-3-3η isoform is expressed at higher levels in patients with arthritis compared with healthy individuals, which is thought to be related to 14-3-3η's direct ability to induce factors linked to inflammation and radiographic damage [8]. 14-3-3η has been shown to induce inflammatory factors such as interleukin (IL)-1 and -6, and is linked to the process of joint damage as it also induces factors such as receptor activator of nuclear factor-kB ligand (RANKL) and matrix metalloproteinase (MMP) 1.

In this study, we analysed the association of baseline 14-3-3η with the development of clinically apparent arthritis in a cohort of subjects with arthralgia who were pre-selected based on being positive for at least one serologic marker: rheumatoid factor (RF) or anti-citrullinated protein antibody (ACPA).

## Methods

### Study participants

From the Reade seropositive arthralgia cohort, the first 144 participants (with ≥30 months of follow-up or development of arthritis, included between 2004 and 2008) were used. This cohort was set up to determine clinical and serological risk factors for development of arthritis, and comprises subjects at risk of arthritis, as defined by arthralgia (no history and no presence of clinically diagnosed arthritis at the time of their first physical examination and no erosions on X-rays of hands and feet) and positivity for at least one serologic marker: ACPA or RF [9].

### Study procedures

At baseline, all participants had clinical and demographic data collected (including visual analogue scale pain, morning stiffness, total of painful and swollen joints) and provided a plasma sample through standard phlebotomy procedures. Enrolment was based on being positive for ACPA and/or RF. Plasma was stored at −20 °C until blinded batch analyses were performed. Following baseline assessments, all participants were re-assessed at regular 12-month intervals over 5 years with emphasis on the development of clinical arthritis. An extra visit could be scheduled in case of arthritis development. Arthritis was defined based on the presence of at least one swollen joint on physical examination of 44 joints by a trained medical doctor (WB or LAS), who was aware of the status of ACPA and RF in the patient. In case of (uncertain) arthritis according to the first observer, the final judgment on presence or absence of arthritis was determined by a senior rheumatologist, who was unaware of the serostatus in the patient (DS). The study was approved by the Ethics Committee of Slotervaart Hospital and Reade, Amsterdam, The Netherlands, and written informed consent was obtained from all study participants.

### Detection of biochemical markers

Baseline plasma was assessed for 14-3-3η levels using the quantitative 14-3-3η enzyme-linked immunosorbent assay (ELISA, Augurex Life Sciences Corp, Vancouver, Canada). Positivity for 14-3-3η was defined as ≥0.19 ng/ml based on the manufacturer's recommended cut-off, and at 2 times and 4 times this cut-off. The development, validation and calibration of the assay are detailed in a recent publication [6]. ACPA was measured by an anti-CCP2 ELISA (Axis Shield, Dundee, UK) and immunoglobulin M rheumatoid factor (IgM-RF) by an in-house ELISA as described previously [10]. The cut-off level for ACPA positivity was set at ≥5 arbitrary units/ml (AU/ml), according to the manufacturer's instructions. The cut-off level for IgM-RF positivity was set at ≥30 international units/ml (IU/ml).

### Statistical methods

The primary outcome chosen was arthritis, not rheumatoid arthritis to prevent circularity, as ACPA and RF are present in the 2010 American College of Rheumatology (ACR)/European League Against Rheumatism (EULAR) RA criteria and 14-3-3η is not. Continuous, normally distributed data were presented as mean (standard deviation) and two-tailed t tests were used to establish whether significant differences existed between groups. Non-normally distributed data were presented as median (interquartile range) and analysed by Mann-Whitney *U* tests. Fisher's exact test was used to identify if positivity for any of the serologic variables investigated (ACPA, RF, and 14-3-3η) was significantly associated with arthritis development over 5 years. Spearman’s rank correlation coefficients expressed the relationship between 14-3-3η and the other serological markers ACPA and RF. Cox-proportional hazards survival analysis tested whether 14-3-3η can predict time to arthritis development.

Generalized linear models (GLM) assessed whether 14-3-3η was independently associated with the development of arthritis within 5 years. We used GLM with binomial outcome and log-link function, rather than standard logistic regression, because of the opportunity to describe relative risks (RR) instead of odds ratios, as this is a more proper association measure for describing results from prospective cohort studies. Since enrolment in the study implied that a subject was either ACPA or RF positive (or both) and no data was obtained in a group negative on both ACPA and RF, we jointly corrected for ACPA and RF status using a categorical variable distinguishing the three groups: (1) only RF positive, (2) only ACPA positive, (3) both RF and ACPA positive. Thereafter we created a variable containing 14-3-3η at different cut-off points (as mentioned above). In the GLM we first put in the categorical variable, after which we added 14-3-3η. The generated *p* values for 14-3-3η can then be interpreted as follows; if significance is found then the 14-3-3η test adds predictive value to the ACPA and RF test in the case one or both of these tests are positive. Note that this significance will imply that the additive value is the same for all three categories. To test whether predictive performance of 14-3-3η depends on the outcome of the ACPA and RF test, we also performed interaction analysis (by adding the interaction between the categorical variable and 14-3-3η in multivariable analyses). This interaction analysis will reveal whether 14-3-3η has more predictive capacity in one of the three groups. All analyses were performed with SPSS version 21 (IBM Corp, Armonk, NY, USA).

## Results

### Arthritis development

Forty-three out of a total of 144 subjects (30 %) developed arthritis after a median of 15 months (Table 1). The median follow-up of subjects not developing arthritis was 60 months (minimum 30 months). Ninety-five percent of the subjects developing arthritis fulfilled the 2010 ACR/EULAR classification criteria for RA [11]. Of those, 28 % fulfilled the criteria regardless of their ACPA and RF serostatus. Five subjects had erosions on their hands or feet X-rays at the time of arthritis diagnosis (out of 36 subjects with X-rays performed). Compared with the subjects not developing arthritis, those that did had significantly more morning stiffness and pain, higher ACPA levels and positivity, and higher 14-3-3η levels and positivity at baseline. Importantly, RF, erythrocyte sedimentation rate (ESR) and C-reactive protein (CRP) were not significantly different between the two groups. At baseline 29 % of subjects used non-steroidal anti-inflammatory drugs (NSAIDs) and no patients received hydroxychloroquine. During the course of the study, 42 % used NSAIDs at one or more time points, and 5 % received hydroxychloroquine (of these patients, five did not develop arthritis whilst two did). Notably, 31 subjects (22 %) received 1–2 dexamethasone injections after baseline in a double-blind trial (which did not delay or prevent arthritis development) [9].

**Table 1:** Baseline characteristics of study participants

Variable	Total group (n = 144)	Arthritis (n = 43)	No arthritis (n = 101)	*p* value
Time until end of follow-up (censoring or arthritis; months)	60 (1–60)	15 (0–60)	60 (30–60)	<0.01
Age (years)*	55 (11)	54 (11)	56 (12)	NS
Males (%)	23	28	21	NS
Disease activity				
Tender joint count 53	0 (0–5)	0 (0–2)	0 (0–5)	NS
Visual analogue scale pain	29 (0–100)	35 (0–100)	26 (0–98)	NS
Use of NSAIDs (%)	29	35	26	NS
ESR (mm/hour)	11 (0–34)	11 (0–34)	11 (1–31)	NS
CRP (mg/l)	2 (0–47)	2 (0–47)	3 (0–27)	NS
Fulfilment of 2010 ACR/EULAR classification criteria for RA (%)	NA	95	0	NA
14-3-3η results				
Level (ng/ml)	0.35 (0.03–20)	0.95 (0.12–20)	0.28 (0.03–20)	<0.01
≥0.19 ng/ml (%)	71	86	64	<0.01
≥0.40 ng/ml (%)	45	58	40	0.04
≥0.80 ng/ml (%)	33	51	24	<0.01
RF results				
Level (IU/ml)	38 (1–1192)	31 (1–383)	40 (1–1192)	NS
Positivity (%)	63	61	63	NS
ACPA results				
Level (AU/ml)	108 (0–9860)	455 (0–8710)	59 (0–9860)	<0.01
Positivity (%)	65	95	53	<0.01

*Abbreviations*: *NS* not significant, *NSAIDs* non-steroidal anti-inflammatory drugs, *ESR* erythrocyte sedimentation rate, *CRP* C-reactive protein, *NA* not applicable, *RA* rheumatoid arthritis, *RF* rheumatoid factor, *IU/ml* international units/ml, *ACPA* anti-citrullinated protein antibodies, *AU/ml* arbitrary units/ml, (*p* value ≥0.05)

*Mean (SD), all other continuous variables mentioned as median (min-max)

### Serological biomarkers 14-3-3η, ACPA and RF

**Fig. 1:**
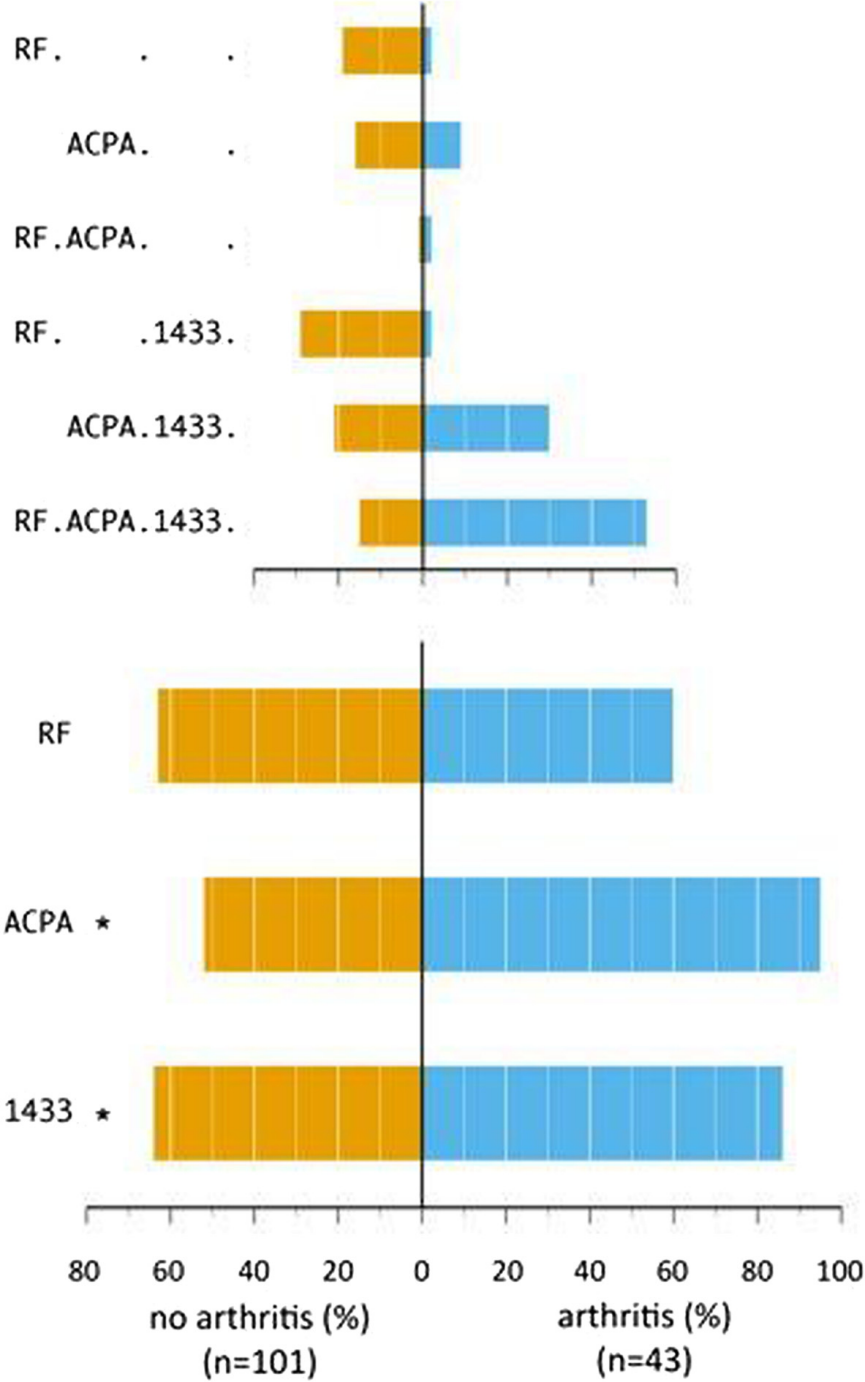
Distribution of positivity for ACPA, RF and 14-3-3η in a cohort of arthralgia patients. *Top panel*: different combinations of positive markers between the subjects developing arthritis versus those who do not; expressed as proportions. *Bottom panel*: percentage of positivity of each marker in the subjects developing arthritis versus those who do not (*Means a statistically significant difference with a *p* value <0.01). *Abbreviations*: *1433* 14-3-3η protein (cut-off ≥0.19 ng/ml), *ACPA* anti-citrullinated protein antibodies, *RF* rheumatoid factor

As represented in Table 1, median 14-3-3η expression levels at baseline were significantly higher in the 43 subjects who developed arthritis in comparison with 101 subjects that did not develop arthritis (median 0.95 vs 0.28, *p* <0.01). Table 1 together with Fig. 1 demonstrate that the prevalence of 14-3-3η positivity at baseline was significantly greater in those patients that developed arthritis in comparison with those that did not at the different cut points (86 % vs 64 %, *p* <0.01; 58 % vs 40 %, *p* = 0.04; 51 % vs 24 %, *p* <0.01 for cut-offs 0.19, 0.4 and 0.8 respectively). Also, the distribution of positivity for ACPA, RF and 14-3-3η and the different combinations between those that developed arthritis and those who did not is outlined in Fig. 1. It shows that subjects developing arthritis were either in the subgroup of ACPA/14-3-3η positives (30 %) or ACPA/RF/14-3-3η positives (52 %). Spearman's rank sum revealed that levels of 14-3-3η were moderately correlated with those of RF and ACPA (0.30 and 0.31, respectively; *p* <0.01). Performance characteristics of 14-3-3η were as follows for the 0.19 cut-off point (manufacturer's recommended cut-off): sensitivity 36 %, specificity 86 %, positive predictive value 86 % and negative predictive value 36 %. Univariate GLM analysis indicated that baseline 14-3-3η positivity significantly predicted arthritis development delivering RRs of 2.5 (*p* = 0.02), 1.7 (*p* = 0.04) and 2.2 (*p* <0.01) at the cut-off points ≥0.19, ≥0.40 and ≥0.80 ng/ml, respectively (Table 2, upper part). GLM evaluating 14-3-3η levels further revealed 14-3-3η's association with the arthritis outcome, with an RR of 1.04 (*p* = 0.01). As previously reported from this cohort, ACPA positivity had a strong association with arthritis development (RR 10.9, *p* <0.01, measured univariately), but RF positivity did not. In multivariable GLM (Table 2, lower part) 14-3-3η levels and positivity at all cutoff points were corrected for the autoantibody status of ACPA and/or RF. Since we used a categorical variable for ACPA and/or RF presence, the generated *p* values for 14-3-3η can be interpreted as predictive capacity of 14-3-3η in the case one or both of these ACPA/RF tests are positive. In this situation, neither 14-3-3η levels nor positivity at any cut-off point added value to the prediction of arthritis development. In the interaction analyses the added value of a positive 14-3-3η test did not differ between subjects that were only RF positive, only ACPA positive or those who were positive for both tests. No significant relation between either 14-3-3η positivity or levels and time of arthritis onset could be found in the Cox proportional hazards model (data not shown). Subgroup analysis of subjects with certain combinations of biomarkers, for example 14-3-3η positivity in ACPA negative versus positive subjects, was not feasible due to small subgroups.

**Table 2:** Univariate and multivariable association of 14-3-3η, ACPA and RF with arthritis development

Univariate logistic regression		
Variable	RR (95 % CI)	*p* value
14-3-3η		
Cut-off ≥0.19	2.5 (1.2–5.6)	0.02
Cut-off ≥0.40	1.7 (1.0–2.8)	0.04
Cut-off ≥0.80	2.2 (1.3–3.5)	<0.01
Levels	1.04 (1.01–1.07)	0.01
Multivariable logistic regression		
Variable	RR (95 % CI)	*p* value
Categorical variable:		
RF	Reference	
ACPA	7.9 (1.9–32.4)	<0.01
RF and ACPA	15.0 (3.8–59.7)	<0.01
Adding 14-3-3η to the above categorical variable		
14-3-3η ≥0.19	1.6 (0.7–3.5)*	0.25
14-3-3η ≥0.40	1.2 (0.7–1.8)	0.52
14-3-3η ≥0.80	1.3 (0.8–2.1)	0.36
14-3-3η levels	1.01 (0.98–1.04)**	0.50

*Abbreviations*: *ACPA* anti-citrullinated protein antibodies, *RF* rheumatoid factor, *RR* relative risk, *CI* confidence interval

*Interpretation of the relative risk, example: subjects that are 14-3-3η positive at the 0.19 cut-off point have a 1.6 times higher risk of developing arthritis, given the knowledge that these subjects must be either RF or ACPA positive

**For continuous variables the relative risk conveys the higher risk of developing arthritis per ascending unit of the independent variable, in this case 14-3-3η

## Discussion

This study presents data that 14-3-3η is present in the pre-clinical phase of arthritis development, since there was a greater proportion of positivity of this marker at study entry together with higher expression in a pre-selected cohort of ACPA- and/or RF-positive arthralgia subjects who developed arthritis, compared with those who did not. This may be related to 14-3-3η’s ability to induce various inflammatory and joint degradative factors [6, 8].

Since 14-3-3η is an inflammatory mediator, the mechanism through which it is related to the development of arthritis may be different from that of autoantibodies such as ACPA and RF, whose levels tend to remain static or unchanged over the course of one’s disease. In this regard, a possible link of 14-3-3η with non-specific measures of inflammation, such as ESR and CRP, might be revealing. However, measurements of ESR and CRP at baseline were related neither to development of arthritis nor to 14-3-3η positivity in this cohort (data not shown). Another difference with autoantibodies might be the dynamic nature of serum 14-3-3η, which was supported by a study in first-degree relatives of indigenous North Americans with RA [12]. This study population was not suitable for serial measurements since half of the patients developed arthritis shortly after inclusion and therefore missed a secondary measurement of 14-3-3η.

In this study, although 14-3-3η was associated with the development of arthritis, baseline 14-3-3η levels and positivity at three cut-off points did not add predictive value to the combination of ACPA and RF. This is most likely influenced by both the pre-selection method for this cohort, the ascertainment of arthritis, as well as the dynamic nature of 14-3-3η. In particular, the blinded confirmatory rheumatologist reviewed only those suspected of developing arthritis, and not all 144 subjects. Since the unblinded physician was making the initial assessment of arthritis development, if a bias did exist from their knowledge of ACPA and RF status, the blinded confirmatory physician would have reviewed a predominance of, say, ACPA-positive subjects and therefore identified more arthritis among those who were ACPA positive.

For clinical practice it would be very useful if 14-3-3η positivity could enhance the prediction of (rheumatoid) arthritis when combined with ACPA and RF. One such study that would enable such an analysis comes from a cohort of subjects based on clinically suspect arthralgia at risk for RA rather than on the basis of positive serology results. In addition to this, a prospective cohort recruited based on the presence of either of the three markers ACPA, RF or 14-3-3η may avoid any underestimation of the predictive capacity of 14-3-3η [13]. Another suggestion would be to use a design which includes serial measurements of 14-3-3η. The OMERACT working group has recommended this design in guidelines to study soluble biomarkers, aimed at clinical validation of their predictive capacity, particularly for prognostic end points [14]. The major limitation of baseline assessment alone has been repeatedly emphasized, particularly for responsive biomarkers such as 14-3-3η, which could vary considerably over the course of disease, and also with therapeutic intervention. This is highlighted in a recent publication describing clinical validation of IL-6 as a predictor of an event where longitudinal, but not baseline assessment alone, was predictive of structural damage in RA [15]. The publication is about progression of radiographic damage, but it applies to other end points as well. However, a single assessment does conform with the clinical situation where a decision is often made to follow the patient or not.

Another limitation of this study was that the primary outcome could not be rheumatoid arthritis, as ACPA and RF are part of the 2010 ACR/EULAR classification criteria and 14-3-3η is not. We explored alternative outcomes such as subjects fulfilling the ACR/EULAR criteria regardless of serostatus and the development of erosions, but not enough subjects were positive for either to allow meaningful analysis.

## Conclusions

In conclusion, we have shown that 14-3-3η is often present in arthralgia subjects positive for ACPA and/or RF prior to the development of arthritis, and was associated with the development of arthritis. In this cohort of subjects pre-selected for ACPA and/or RF positivity the added predictive value of 14-3-3η, both levels and different cut-off points, could not be established. Further studies are warranted to assess the combined utility of these three markers in predicting the development of arthritis.

## Abbreviations

ACPAs: anti-citrullinated protein antibodies
anti-Carp: anti-carbamylated protein antibodies
anti-PAD-4: peptidyl arginine deiminase type 4
AU/ml: arbitrary units/ml
CI: confidence interval
CRP: C-reactive protein
DS: Dirkjan van Schaardenburg
ELISA: enzyme-linked immunosorbent assays
ESR: erythrocyte sedimentation rate
GLM: generalized linear model
IgM-RF: immunoglobulin M rheumatoid factor
IL: interleukin
IU/ml: international units/ml
LAS: Lotte van de Stadt
MMP: matrix metalloproteinase
NSAIDs: non-steroidal anti-inflammatory drugs
RANKL: receptor activator of nuclear factor-kB ligand
RA: rheumatoid arthritis
RF: rheumatoid factor
RR: relative risk
WB: Wouter Bos
